# Motion tracking of iris features to detect small eye movements

**DOI:** 10.16910/jemr.12.6.4

**Published:** 2019-04-05

**Authors:** Aayush K. Chaudhary, Jeff B. Pelz

**Affiliations:** Carlson Center for Imaging Science, Rochester Institute of Technology, NY, USA

**Keywords:** Microsaccades, eye movements, eye tracking methodology, iris features, visual fixations, video-based eye tracking, head motion compensation, iris segmentation

## Abstract

The inability of current video-based eye trackers to reliably detect very small eye movements has led to confusion about the prevalence or even the existence of monocular microsaccades (small, rapid eye movements that occur in only one eye at a time). As current methods often rely on precisely localizing the pupil and/or corneal reflection on successive frames, current microsaccade-detection algorithms often suffer from signal artifacts and a low signal-to-noise ratio. We describe a new video-based eye tracking methodology which can reliably detect small eye movements over 0.2 degrees (12 arcmins) with very high confidence. Our method tracks the motion of iris features to estimate velocity rather than position, yielding a better record of microsaccades. We provide a more robust, detailed record of miniature eye movements by relying on more stable, higher-order features (such as local features of iris texture) instead of lower-order features (such as pupil center and corneal reflection), which are sensitive to noise and drift.

## Introduction

To maintain vision and compensate for the intersaccadic drift between large eye movements, humans continuously make small (miniature) eye movements including tremor, drift, and microsaccades [ 1, 2, 3 ] . These movements occur even when we try to fixate on a point in the world. Low-frequency oscillations of the eyes, unsteadiness of the oculomotor system or attempts to maintain visual perception might result in the production of these movements [ 4, 5 ] . Among these small eye movements, microsaccades have been an area of interest in the community because of their vital importance in restoring perception and maintaining vision [ 6, 7, 8, 9, 10 ] , medical diagnosis [ 11, 12, 13 ] and their ability to indicate attentional shifts [ 14, 15, 16 ].

Microsaccades are the small, jerk-like motions [ 17 ] which are generally found to co-occur in two eyes [ 4, 18, 19, 20 ]. While the term ‘microsaccade’ is sometimes used only for involuntary eye movements that occur during fixation (e.g., [ 17 ] ), we adopt the less restrictive definition of a microsaccade as any rapid eye movement with an amplitude below 0.5 degrees of visual angle. Microsaccades and saccades follow the same *main sequence* [ 21 ] and have a common generator [ 22 ] . Following previous work (e.g., [ 10, 14, 23, 24 ] ), we regard both voluntary and involuntary saccades below 0.5° as *microsaccades*.

Currently, there is disagreement in the literature about the presence of monocular microsaccades during everyday vision. Gautier et al. [ 25 ] has argued that the practical role of monocular microsaccades may be to aid vergence and make accurate corrections of eye position. However, recent publications [ 26, 27 ] have questioned the existence of such eye movements and raised concerns about previously published results, suggesting that the majority of monocular microsaccades may be measurement artifacts.

One of the limitations of the current generation of video-based eye trackers is their inability to precisely detect microsaccades because the size of those eye movements is similar to the noise level of the trackers which rely on precisely localizing the pupil and/or corneal reflection (CR) on successive frames. Moreover, some of these methods use only pupil information to acquire a higher sampling rate, but in these methods, relative motion between the head and camera are not taken into account [ 28 ] , which can increase the false detection of microsaccades. Fang et al. [ 26 ] and Nyström et al. [ 27 ] analyzed many small eye movements that were classified as monocular microsaccades by such algorithms and suggested that most of those events were binocular events or noise. Fang et al. [ 26 ] adjusted the threshold parameter to classify them correctly as binocular microsaccades whereas Nyström et al. [ 27 ] used manual inspection for verification.

New algorithms that take advantage of modern high-resolution cameras and recent advances in computer vision can overcome some of these issues. High-resolution cameras make it possible to extract fine local features such as iris textures. Using populations of such features in place of large, single features such as the pupil allows for a higher degree of confidence in microsaccade detection. Additionally, tracking the motion of those features across consecutive frames allows for the study of motion distributions, simplifying the analysis of small eye movements. We demonstrate that microsaccades less than 0.20 degrees of visual angle can be reliably detected with these techniques and a new velocity tracking algorithm. The method is validated with two types of experimental tasks; reading characters on a test target and watching videos.

## Methods

**Figure 1.**
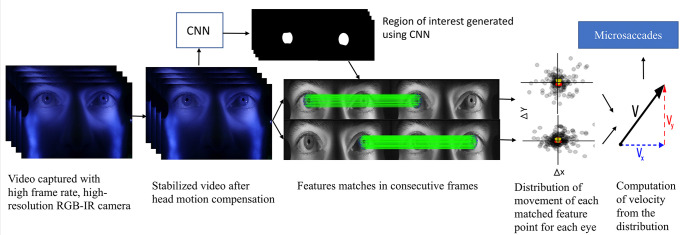
Block diagram of the overall system.

Eye movements are monitored by extracting the motion vectors of multiple iris features. A high frame-rate, high-resolution camera is used to capture a video of an observer’s face containing the eyes and surrounding regions. A trained convolutional network (CNN) [ 29 ] is used to segment the iris from each frame to extract features only from the region of interest. To ensure high-quality motion signals, Speeded Up Robust Features (SURF) feature descriptors [ 30 ] are used to extract feature vectors which are then matched in consecutive frames using brute force matching followed by random sample consensus (RANSAC) [ 31 ] and homography [ 32 ] .Tracking the geometric median of these matched keypoints excludes outliers, and the velocity is approximated by scaling by the sampling rate [ 33 ] . Microsaccades are then identified by thresholding the velocity estimate. For head motion compensation across each frame, a hybrid cascaded similarity transformation model is introduced using tracking and matching of features of rectangular patches in regions of the observer’s cheek. The overall system block diagram is shown in Figure 1.

### Iris Segmentation

The region of each frame containing the iris must be isolated from the rest of the frame before velocity estimation can begin. A number of approaches have been used for iris segmentation including ellipse fitting, geodesic active contours [ 34 ] , Hough circle fitting, edge detection, integrodifferential operators [ 35 ] , graph cuts [ 36 ] , and Zernike moments [ 37 ] . All of these methods require tuning for good results and fail to generalize across observers with different iris and skin pigmentation. A trained CNN makes it is possible to generate a more robust solution across observers.

**Figure 2.**
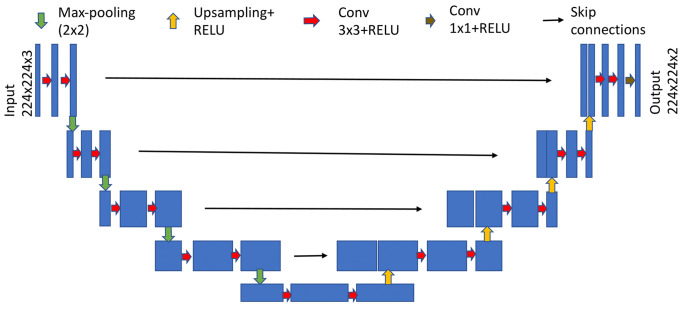
Segmentation model based on U-Net architecture after [38].

**Network Architecture.** A U-Net based architecture [ 38 ] allows for segmentation of the eye region with a moderate training set. The model combines the localization and context information using contracting and expanding paths. The contracting path consists of sequences of two blocks of a 3x3 convolution layer followed by a rectified linear unit (ReLU) as non-linear transformation with batch normalization, then a 2x2 max-pooling operation. The expanding path follows sequences of upsampling with a scale factor of two and then concatenating with its subsequent feature map from a skip-connected layer. This upsampling block is followed by two blocks of 3x3 convolution layers and later by activation of ReLU with batch normalization. The model achieves good performance and is best suited for biomedical applications with limited data [ 38 ] . Figure 2 shows the segmentation model.

**Figure 3.**
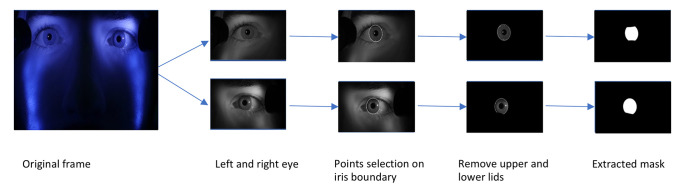
Procedure used for labelling the train and test sets.

**Data labelling.** Instead of training on the full-resolution (1920x1080) video, each frame was partitioned into three regions: left and right eye (960x540 each) and the lower part of the face (960x1080). Binary labels of iris and non-iris regions were generated for training the model by manually clicking six – ten points on the border of the iris on each image as shown in Figure 3, and an ellipse was fit to those points with a least square ellipse fitting method [ 39 ] . Since the generated ellipse often overlapped portions of the eyelids, points were also selected along the border with the upper and lower eyelids and used to fit second-degree polynomials to produce the final ground-truth iris regions. The training set contained a total of 406 images from video frames of four observers. The training set was augmented by flipping each image horizontally, producing a total of 812 images. The testing set consisted of 260 images of correlated data (training and test set from the same observers) and 126 images of uncorrelated data.

**Training procedure.** Training images were re-sized to 224x224. The R, G, and B channels were used as input to the model for the desired two (iris and non-iris) output classes. The Adam Optimizer [ 40 ] was applied for regularization with a learning rate of 0.0001 with an exponential decay rate for the first moment estimate of 0.55, and an exponential decay rate for the second moment estimate of 0.99. The models were run in Pytorch [ 41 ] on an Ubuntu 16.04 LTS platform on a Nvidia Titan 1080 Ti. The training was done with a batch size of eight samples (limited by GPU memory), and the model was run for 40 epochs.

**Performance Metrics** Pixel-wise cross entropy of class probabilities was used during training as a loss function. The accuracy of iris detection was measured with the Intersection over Union (IoU) metric. The IoU metric varies from 0 – 1 and is defined as the ratio of the intersection of the ground-truth and predicted regions to the union of those regions [ 42 ] . Perfect agreement between ground-truth and prediction yields 1; no overlap yields 0.

**Figure 4.**
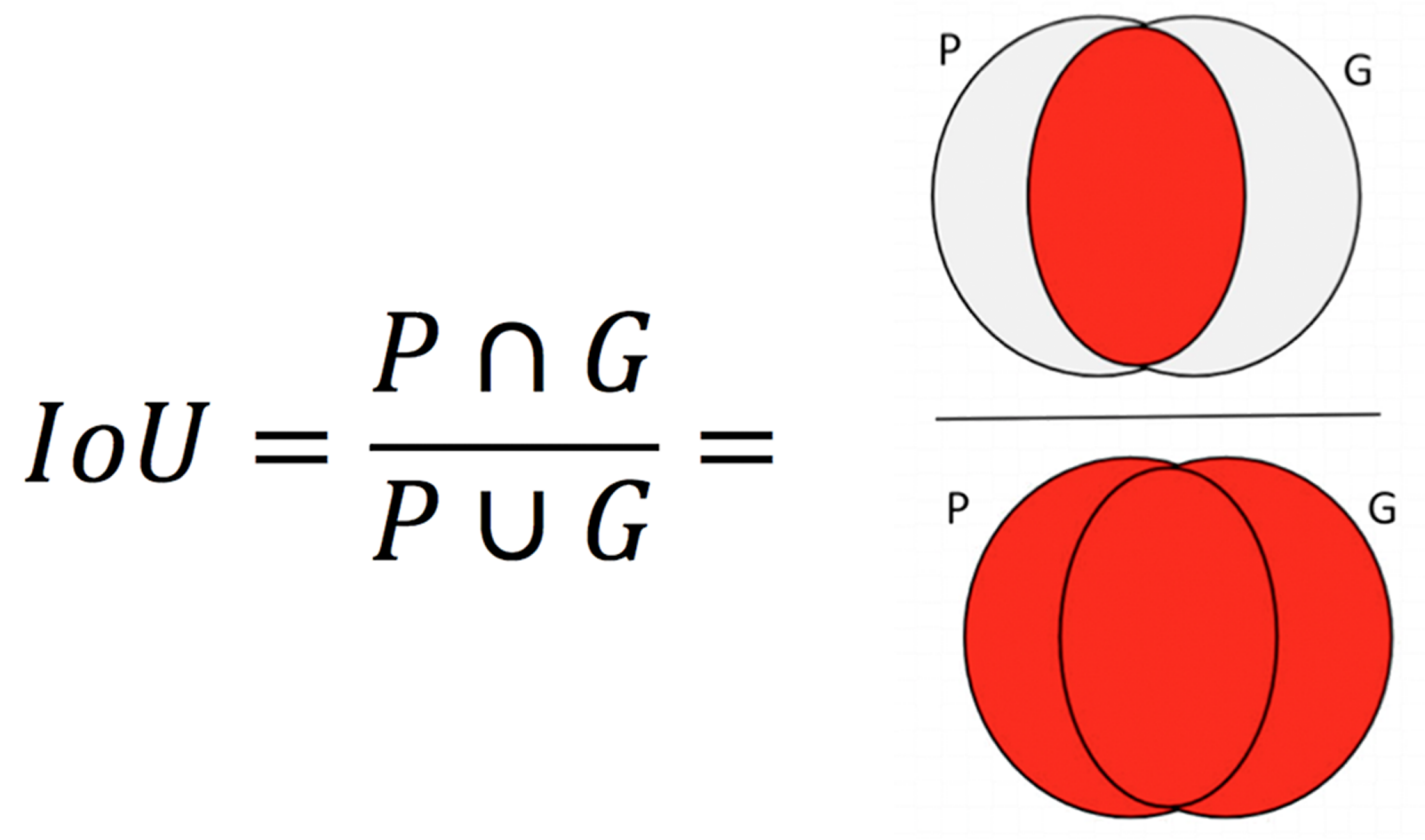
IoU is computed as the ratio of intersection over union. The overlapping regions and non-overlapping regions are indicated by red and gray colors respectively.

If P is the predicted label and G is the ground truth, IoU is computed as shown in Figure 4.

### Head-motion compensation

Even when an observer’s head is stabilized by a chin rest, head movements on the scale of small eye movements still occur, so it is crucial to compensate for this motion when detecting microsaccades. Complex solutions using 3D head models or external hardware trackers could be used, but in this work, we propose an image-based method that compensates with a simple planar image transformation based on regions detected on the face.

**Figure 5.**
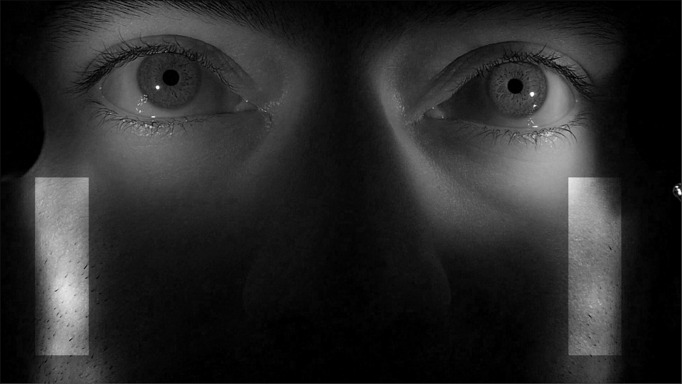
Indication of regions (light patches) on face tracked along with iris regions for head-motion compensation

The human face is non-planar and deformable. The eyes are not necessarily in the same plane as the nose, cheek or forehead, making planar transformation models like homography (8 degrees of freedom (DOF)), affine (6 DOF) or similarity (4 DOF) transforms imperfect approximations. Here, we make the simplifying assumption that the regions selected in Figure 5 fall approximately in the same plane as that of the two irises being tracked. Relatively planar regions that do not move with normal eye movements were manually selected for each subject.

Head movements were compensated by automatically aligning each frame of a video to the initial frame of that video. Computing the homography transformation between frames based on good feature matches across adjacent frames allows a sequence of frames to be aligned. A list of good matches between pairs of adjacent frames was determined with Lowe's ratio test [ 43 ] followed by outlier removal with RANSAC for matched features between consecutive frames. The matched features were found by extracting the local features in the selected regions using SURF and then by using the brute-force matcher where the descriptor of each feature in the nth frame was matched with descriptors of all features in the (n+1) ^th^ frame using the L2 distance norm.

Motion compensation can be accomplished for an entire video by aligning each frame in this way to the initial reference frame by cascading the homography transformations as proposed by Dutta et al. [ 44 ] . We found, however, that this approach aggregated skew and perspective errors across frames because the points on which computation of the homography matrix was based were not consistent within each region. Because there tended to be more texture in the regions of the lower cheek than near the eyes even though they were in approximately the same plane, more of the features used for tracking were from the cheek regions. Constraining the feature path to a rectangle as in Grundmann et al. [ 45 ] decreased errors but did not provide an acceptable solution.

**Figure 6.**
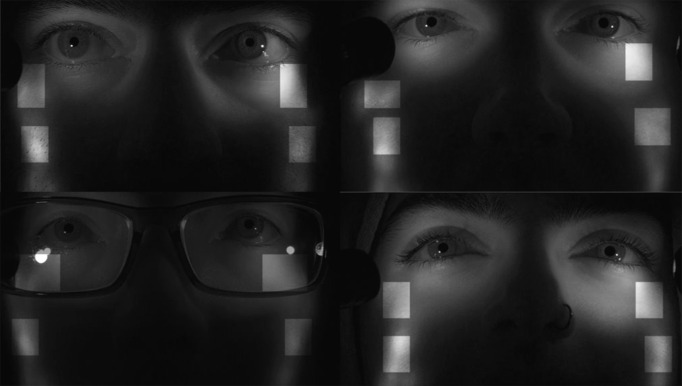
Regions (light patches) on four observers faces used for headmotion compensation.

Instead, we selected features in rectangular regions as seen in Figure 6, then computed good matches between consecutive frames for each of the regions. Computation of the geometric median of those matches provided an estimate of the best value of the distribution of the rectangular patch in the source and destination images. Using one point from each of the four regions we computed the transformation between image pairs. The homography transformation is the least-constrained plane-to-plane mapping, with eight degrees of freedom (DOF) which can be described as allowing translation (2 DOF), scaling (2 DOF), rotation (2 DOF), and keystoning (magnification varying by position; 2 DOF). Other transformations are more constrained. The affine transform, for example, allows only six degrees of freedom which can be described as translation (2 DOF), scaling (2 DOF), rotation (1 DOF), and shearing (translation varying by position; 1 DOF). The similarity transformation has only four degrees of freedom, allowing only translation (2 DOF), uniform scaling (1 DOF), and rotation (1 DOF). As shown by Grundmann et al. [ 45 ] , the extra degrees of freedom of the homography and affine transformations can result in larger errors than the more constrained similarity transformation, but we found that skew errors still grew over time even with the similarity transform.

Dutta et al. [ 44 ] identified *breaks* in a video sequence when the video starts panning or scale changes become substantial. Information from prior frames is not considered after a *break* and a new reference frame is introduced. Grundmann et al. [ 45 ] proposed a different approach in which all the keyframes are matched, and transformation between the keyframes is initially computed. Then, a similarity transformation is found from the starting keyframe to each subsequent frame until the next keyframe. A small jitter is observed when moving from the final intermediate frame to the next aligned keyframe, so there is a tradeoff in the frequency of re-aligning with a new keyframe; more frequent realignment minimizes the size of the jitter but increases its frequency.

**Figure 7.**
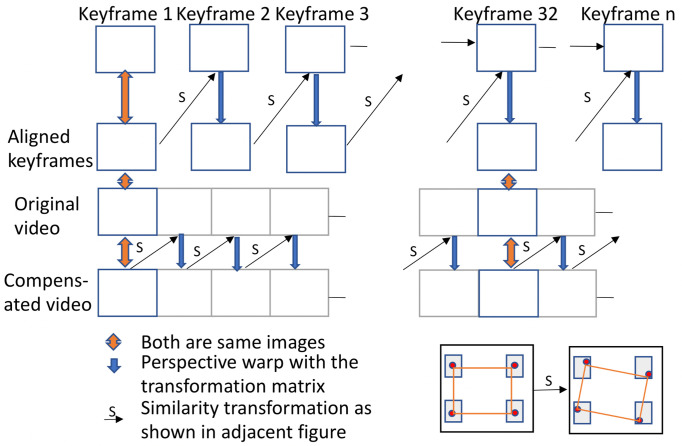
Model used for head motion compensation.

We elected to realign to a new keyframe once every 480 frames (5 s). Less frequent realignment increased the aggregated skew and perspective errors, and more frequent realignment increased the frequency of the jitter. The overall model is shown in Figure 7.

### Velocity approximation

After the segmentation of irises in the stabilized video, the next crucial step is to extract the iris features. These features were extracted in a Contrast Limited Adaptive Histogram equalized grayscale representation of the image [ 46 ] as this resulted in a larger number of good matches across different observers than did any single-color channel. From the extracted features across consecutive frames, good matches were found using brute-force matching followed by Lowe's ratio distance test and outlier removal by RANSAC. The motion of the iris between adjacent video frames was represented as a motion vector.

These motion vectors can be used to compute horizontal, vertical and torsional eye movements. A number of researchers have used iris features to measure torsional eye movements [ 47, 48, 49 ] . Ong and Haslwanter [ 49 ] transformed the image of the iris into polar coordinates about the pupil center so that torsion could be tracked as translation along the angular axis.

Because we are concerned with microsaccades in this work, we ignored torsional movements and computed the geometric median of the shift of all matched keypoint pairs between frames. The relative velocity of each eye was calculated independently as the product of the scaled motion vector and the sampling rate, and integrating those values gave a relative position signal for each eye. Calibration was performed by simple linear regression of the relative position signal and the known gaze position during calibration. Position cannot be estimated during blinks, so the data reported here include only trials completed without blinks during the calibration routine.

The raw signal was filtered using 1D total variation denoising (TVD) [ 50 ] which minimizes the variation as a least squares problem. Instead of computing an estimate on a local neighborhood, TVD uses the entire record to estimate a global optimum [ 51 ] to minimize undesired spurious noise while preserving the saccadic ‘edges.’ TVD is preferable to smoothing algorithms because our goal was to model the sensor noise rather than eye position noise using the overall signal. The key parameter in the TVD filter is a regularization parameter which was set to 0.1 for all observers. After denoising, cyclopean gaze velocity was computed from the right and left eye velocities.

### Microsaccade detection

A number of methods have been proposed for microsaccade detection [ 14, 52, 53, 54 ] . Almost all of the algorithms have a tunable parameter that affects algorithm performance. We implemented an adaptive version of the Velocity-Threshold Identification algorithm (I-VT) [ 55 ] . Here, microsaccades were detected by thresholding the generated absolute cyclopean velocity signal. Determining the velocity thresholding parameter is an essential step in categorizing events, as a high threshold would increase the miss rate, and a low threshold would increase false alarms. A fixed threshold did not work well for all observers because of individual variations, noise in the system due to prevailing signal artifacts and small, uncompensated head movements.

**Figure 8.**
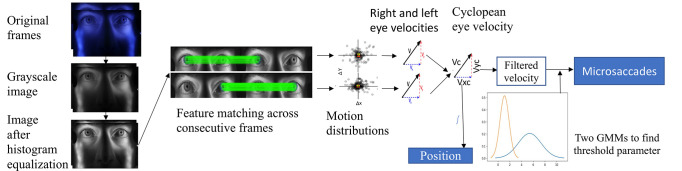
Block diagram of iris feature extraction to microsaccades detection.

To automatically adapt to individual observers, we implemented an adaptive-threshold method based on a Gaussian mixture model (GMM) with two velocity distributions representing noise and microsaccades. To eliminate larger saccades, only absolute velocities below 20 deg/sec were passed to the model. A thresholding parameter based on estimated mean and standard deviation using 99.7% of each distribution was used. Based on observations of the noise floor, a lower bound of 3.84 deg/sec was selected to minimize false alarms. Figure 8 shows the block diagram from the iris feature extraction to microsaccade detection.

## Experimental Setup

**Figure 9.**
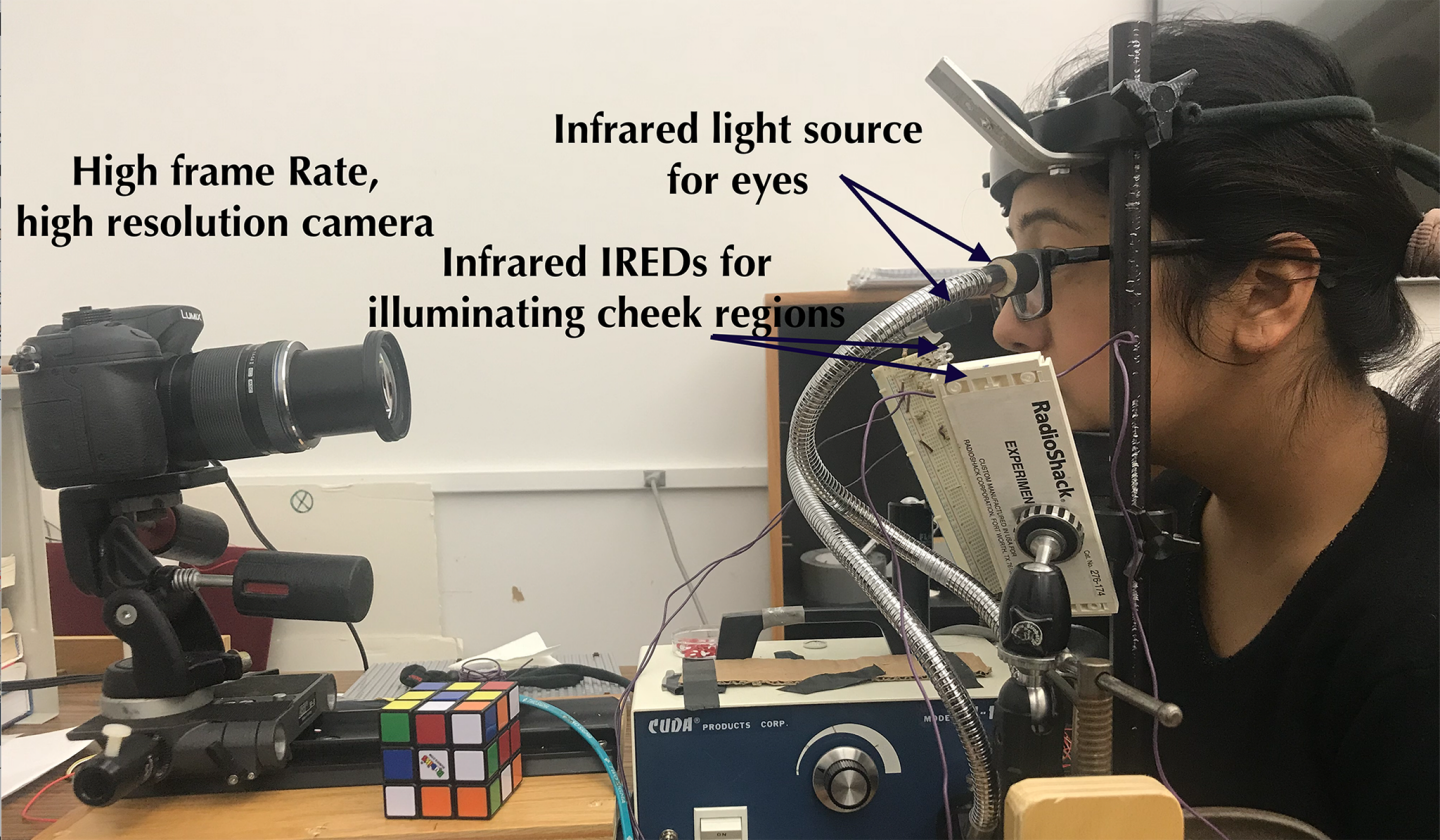
Experimental setup with calibration targets and the scene behind the camera.

Binocular eye movements were recorded using a Panasonic Lumix DMC-GH4 mirrorless digital camera modified by removing the IR-rejection filter. Video recordings of the eyes and the region surrounding the eyes were recorded at a frame rate of 96 frames per second (fps). Head movements were restricted with a UHCOTech HeadSpot chin and forehead rest.  Filtered tungsten and LED infrared light sources were used to illuminate the eyes and the region surrounding the eyes as shown in Figure 9. Subjects were asked to sit comfortably to minimize head movements. Trials were repeated until calibration was completed without blinks.

**Apparatus.** The Lumix DMC-GH4 was set to ISO 400, and F/8. The camera was placed at a distance of 50 cm from the observer, and the center of the lens was positioned 4.5 cm below the eyes at an angle of approximately 4 degrees with the horizontal axis (the frame was not centered on the eyes). A focal length of approximately 70 mm was used to capture an appropriate region including the eyes and surrounding areas.

**Light source. **The experiments were conducted in a lab with indirect fluorescent illumination. A tungsten-halogen source with a bifurcated fiber-optic light guide was filtered with a near-infrared high-pass (750nm) filter. As seen in Figure 9, the light guides were placed at an angle ~25-30 degrees above the horizontal, separated by 88mm. The sources provided an irradiance of 0.020 – 0.037 W/cm [ 2 ] at the iris. Each side of the observers’ cheek regions was illuminated with twelve 940 nm infrared emitting diodes (IREDs) (Vishay VSLY5940) adjusted to match the exposure of the iris. The IREDs were arranged in parallel lines, separated by 7 mm, and placed to the side of the observer so that the topmost IREDs were approximately 5 cm below the eyes.

### Subjects

Eye movements of seven participants (5 males, 2 females) with normal or corrected-to-normal vision were recorded. Participants with light and dark irises and with and without prescription glasses were selected. Subjects were undergraduate and graduate students with a mean age of 25 years (σ=3). The experiment was conducted with the approval of the Institutional Review Board and with the informed consent of all participants.

### Tasks

Each observer performed three tasks, each preceded by a task-specific 9-point calibration routine.

#### Snellen microsaccade task

Microsaccades can be evoked when observers read small, isolated characters, as in the Snellen eye chart [ 56 ] . Calibration points and a ‘pocket’ Snellen chart (7″, x 4″, designed for use at 14″) were placed at a distance of 150 cm from the observer 3 cm above the center of the subject's eyes. The field of view of the calibration target was 8.7° x 6.9°. The subjects were asked to fixate each point in the calibration target, then look at each character on a line of the Snellen chart at a distance of 150 cm. Each character subtended a vertical angle of approximately 5 arcminutes (equivalent to a ‘20/20’ character at that distance). The subtended horizontal angle between eight characters in the chart was approximately 0.15 degrees; two others were 0.28 and 0.32 degrees. The small eye movements evoked as observers looked at the characters on the Snellen eye chart which were used to verify the detection of microsaccades using our algorithm.

#### Video Stimuli

To examine the detection of microsaccades while viewing a moving stimulus, observers viewed two short videos from [ 57 ] on a 12-inch Apple MacBook (MF855LL/A). In the first video, the observers were instructed to track the motion of a car turning in a tight *figure-8* pattern. In the second video, they were instructed to track the cup containing a marble in a ‘shell game.’ Both videos induced smooth pursuit eye movements. The laptop was placed on a table so that the display center was 100 cm from the observer and 10 cm above the subject’s eyes.  The display field of view was 13.5° X 7.4°.

## Results

### Segmentation Results

**Figure 10.**
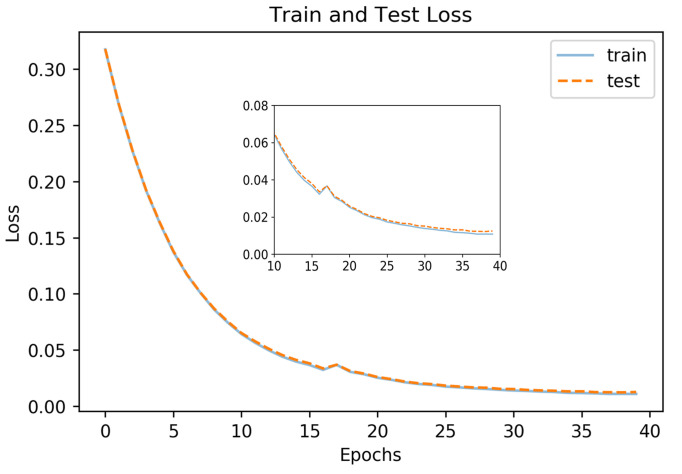
Loss curve for segmentation model.

Figure 10 shows the loss function for the training and the testing datasets. The model starts to reach its asymptote at 35 epochs for both sets. For our model, the average IoU values were 0.898 for the training set, 0.891 for the correlated test data, and 0.866 for the uncorrelated test data.

**Figure 11.**
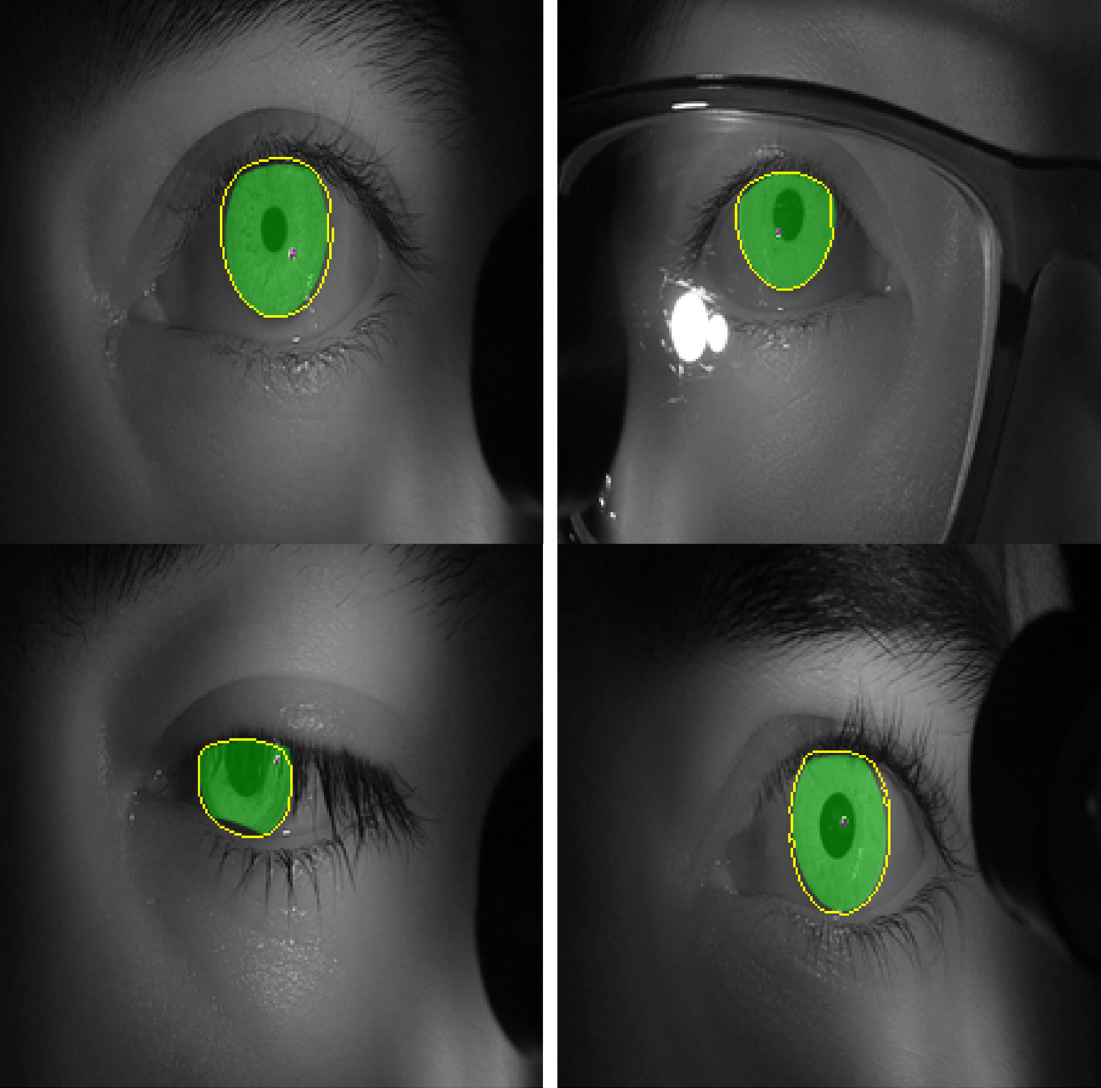
Images of test set with labels predicted by the model in yellow color (edges) and the labelled ground truth in green color (filled).

Figure 11 shows images with iris regions predicted by the model and ground truth indicated in yellow and green colors respectively. For proper visualization, only the border predicted by the model is shown. The large white regions in the upper-right image are specular reflections in the observer’s eyeglasses. A spike in the loss function at epochs 16-17 evident in Figure 10 is because of inconsistency in some of the labeled data such as when the eyelids cover the iris as seen in the lower left image in Figure 11.

### Video Stabilization

**Figure 12.**
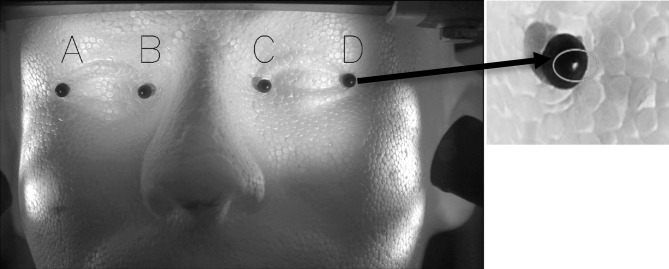
Face model used for head stabilization verification with markers A, B, C and D indicated in the figure (Left). (Right) The circle indicates the bright spot used for our method verification

**Figure 13.**
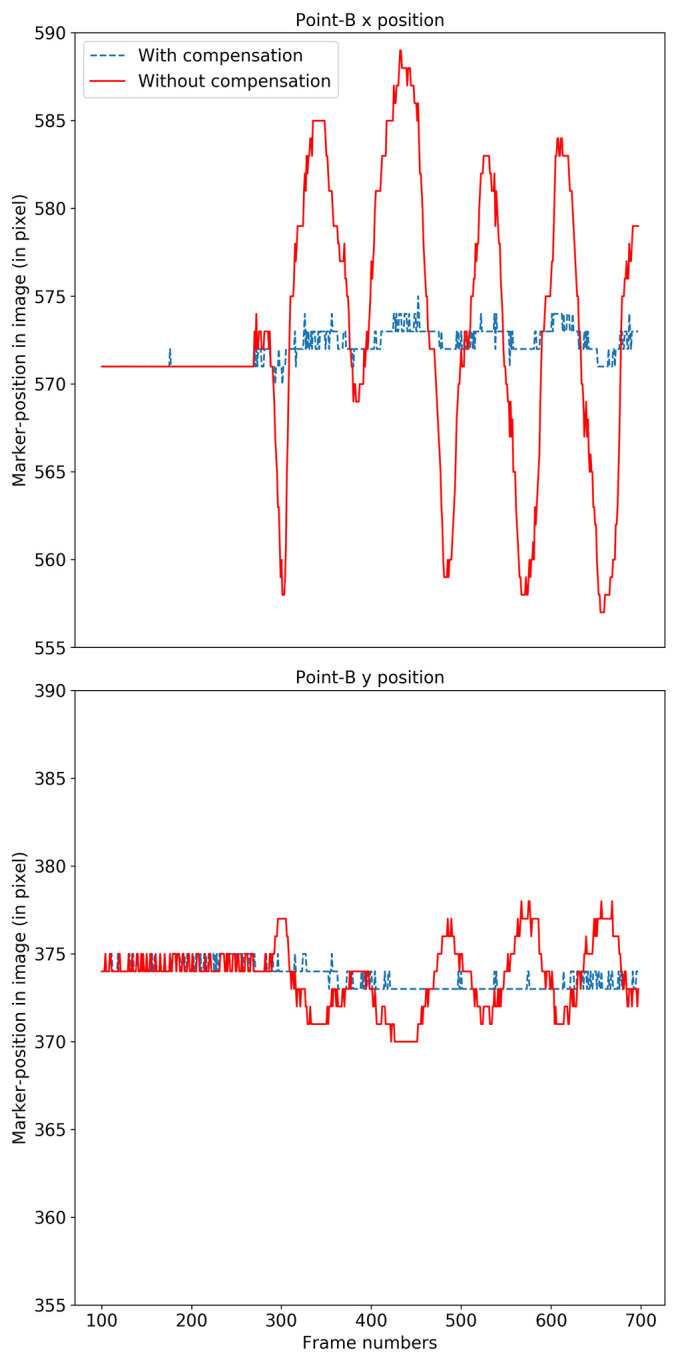
The motion of the original face model (red) and after compensation (blue) for point B is shown in the x-direction (top) and y-direction (bottom).

We evaluated the video stabilization for two cases; a rigid Styrofoam model head and for real human faces. For the rigid head model, we tracked the four points labeled A, B, C and D in Figure 12 (left). The rigid head was rotated slightly about the base, resulting in horizontal, vertical and diagonal movements of approximately 2 mm at a frequency of approximately 1 Hz, and the video was stabilized using our compensation model. The original motion for point B is shown in solid red in Figure 13 and the motion-compensated output is shown in dashed blue.

Table 1 shows the mean squared change (MSE) from the initial starting reference frame and standard deviation (STD) of points before and after stabilization in pixels. The overall mean per class (MPC) across all points shows a dramatic improvement in performance. The test points were tracked by locating the brightest spot (a specular reflection) on the black marker as indicated in Figure 12 (right). A variation of one pixel is expected because the maximum location algorithm in OpenCV [ 58 ] returns the horizontal and vertical pixel location as integer values.

**Table 1:** Motion before (without compensation) and after (with compensation) results for each point and overall mean per class (MPC).

Points	Before		After	
	MSE	STD	MSE	STD
A x	5.28	7.19	0.48	0.65
A y	2.62	3.58	0.44	0.51
B x	5.38	7.37	0.61	0.79
B y	1.31	1.8	0.21	0.44
C x	5.53	7.57	0.52	0.76
C y	0.77	1.16	0.51	0.56
D x	5.56	7.64	0.42	0.66
D y	2.26	2.92	0.21	0.44
MPC	3.59	4.9	0.42	0.6

We also examined the performance of the video stabilization algorithm for a seated observer in a chinrest looking at nine calibration points for about a second each. Head motion was tracked by attaching two small stickers to the observer’s face and tracking each sticker in the video by computing the mean of the central votes of matched features by using consensus-based matching and tracking [ 59 ] . Before and after compensation results are shown in Table 2. Unlike the rigid head model, where intentional movements were introduced, we expected only minimal head movements in the human face condition. The result shows an improvement in most of the cases, but in one case (S2), the standard deviation increased along the x-axis by approximately 20% while decreasing along the y-axis by 32%.

**Table 2:** Standard deviation (in x and y pixel position) of movements of two points in either side of face in the original video and after compensation.

Subject	Sticker placed on	Original		After	
		STDx	STDy	STDx	STDy
1	Left	2.2	1.22	1.05	1.11
	Right	1.45	1.37	1.12	1.03
2	Left	0.8	3.15	0.88	2.23
	Right	1.02	2.49	1.24	1.71
3	Left	0.98	1.67	1.26	1.51
	Right	1.46	2.29	0.57	1.08
4	Left	2.78	5.08	2.9	2.21
	Right	3.11	4.53	1.39	2.24

### Snellen microsaccade task

**Figure 14.**
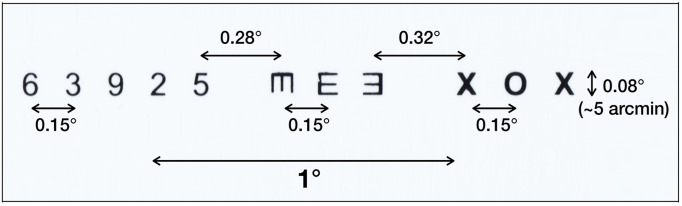
Target line from pocket Snellen chart. Characters in three groups subtended a vertical angle of 5 arc minutes.

**Figure 15.**
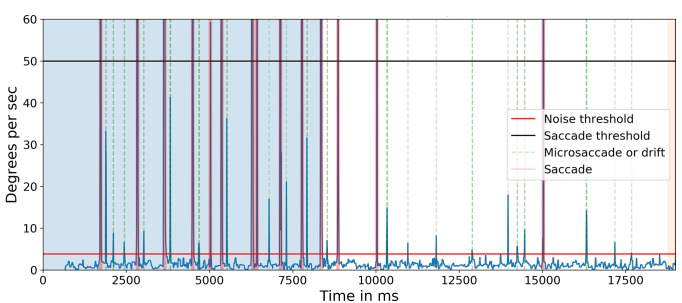
Absolute velocity (deg/sec) of the cyclopean gaze. Light green shows the small eye movements detected between 3.84 deg/sec to 50 deg/sec. There should be at least eight 0.15-degrees, one 0.28-degrees, and one 0.32-degrees, a total of 10 movements after 8500ms (in the non-highlighted region) as targets in one line of the Snellen chart. The initial 8500ms are the calibration phase.

In the first task, observers were instructed to read a line on the pocket Snellen eye chart whose characters subtended an angle of approximately 5 arcminutes (equivalent to a ‘20/20’ character at that distance). Because of the size and distance of the target, the eye movements necessary to foveate each target constitute microsaccades [ 56 ] . Figure 14 shows the target and the angular subtense of the microsaccades required to move between the characters in each of the three groups (0.15°) and between the two groups (0.28° and 0.32°). Figure 15 shows the events detected for one of the subjects. The blue line indicates the cyclopean eye absolute velocity; the green dashed lines indicates the microsaccades or drifts detected which is a superset of the fixation targets. These microsaccades were detected with an absolute velocity threshold of 3.84 deg/sec. Note the clear separation between the signal and noise. Red lines indicate saccades with an absolute velocity greater than 50 deg/sec. The saccadic movement that appears every 5000ms is an artifact of the jitter caused when a new keyframe is taken as a reference during head stabilization.

**Figure 16.**
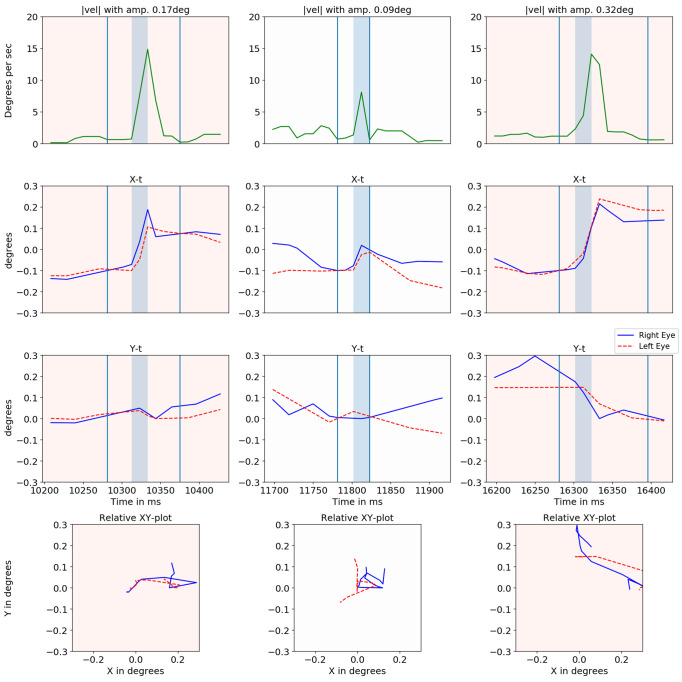
Cyclopean velocity plot, relative X-t, Y-t and XY plot for right eye (blue) and left eye (dashed red) is shown for three events in different columns.

Figure 16 plots the absolute velocity of the cyclopean gaze (with its amplitude), X-t, Y-t and YX graphs for both eyes when microsaccades were detected. The amplitude of each microsaccade was calculated based on the gaze position between the local minima of the cyclopean velocity before and after the microsaccades. The two local minima are indicated in the absolute velocity plot by the vertical light blue lines. There is a close relationship between the two eyes for most of the cases. The center column shows a case where the eyes were moving in opposite directions.

Results for all seven subjects are shown in Table 3. The table shows the number of microsaccades per observer: (A) found when the eye video was inspected visually; (B) detected by the algorithm; (C) detected by the algorithm when separated by an interval equal to at least half of the average duration available for each character; and (D) detected by the algorithm but not present (‘false alarms’).

**Table 3:** Number of microsaccades detected manually by inspecting video and using the algorithm for all subjects. The total number of instructed targets is 8 targets of 0.15 degree and 2 targets over 0.2 degrees. The table also shows number of false alarms observed.

Subjects			1	2	3	4	5	6	7
0.15 degrees (out of 8 instructed targets)	(A) Microsaccades observed by inspecting video		8	8	3	14	19	15	10
	Algorithm	(B) Detected	6	8	3	16	18	12	10
		(C) Finite interval gap (out of (4,2,2) events)	(2,1,2)	(4,1,2)	(2,1,0)	(4,0,5)	(5,1,6)	(3,6,2)	(3,3,1)
(D) Number of false alarms			0	0	0	2	1	2	0

The events for 0.15 degree microsaccades in Table 3 (C) should be (4, 2, 2) as the instructed targets are set in that order as seen in Figure 14. Initially, two events of 0.28-degree and 0.32-degree microsaccades are determined. Then, the number of events before 0.28-degree microsaccades, between 0.28 degree and 0.32 degree and after 0.32 degree are detected with a consideration that only one unique microsaccade must be a voluntary movement made for the target in the interval. All the other movements in the interval are neglected as our interest lies in detecting movements for the instructed target rather than observing the number of overall detected microsaccades. Note that the method identified all small movements ≥ 0.2 degrees.

Identifying small movements with a magnitude of 0.15 degree is possible for some events when a lower threshold is selected, but this increased the false alarm rate. Subjects 5 and 6 were two cases of noisy data. For Subject 6, using an initial regularization parameter of 0.1 for filtering resulted in eight false alarms and a miss rate of 11.1% (with respect to the total number of microsaccades observed by inspecting video). It was seen that signal was noisy even after head motion compensation thus changing filtering regularization parameter to 0.20 and 0.15 for the right and left eye respectively there was an improvement in the result with only two false alarms. However, higher values of filtering regularization parameter increased the miss rate to 33.3%.

**Figure 17.**
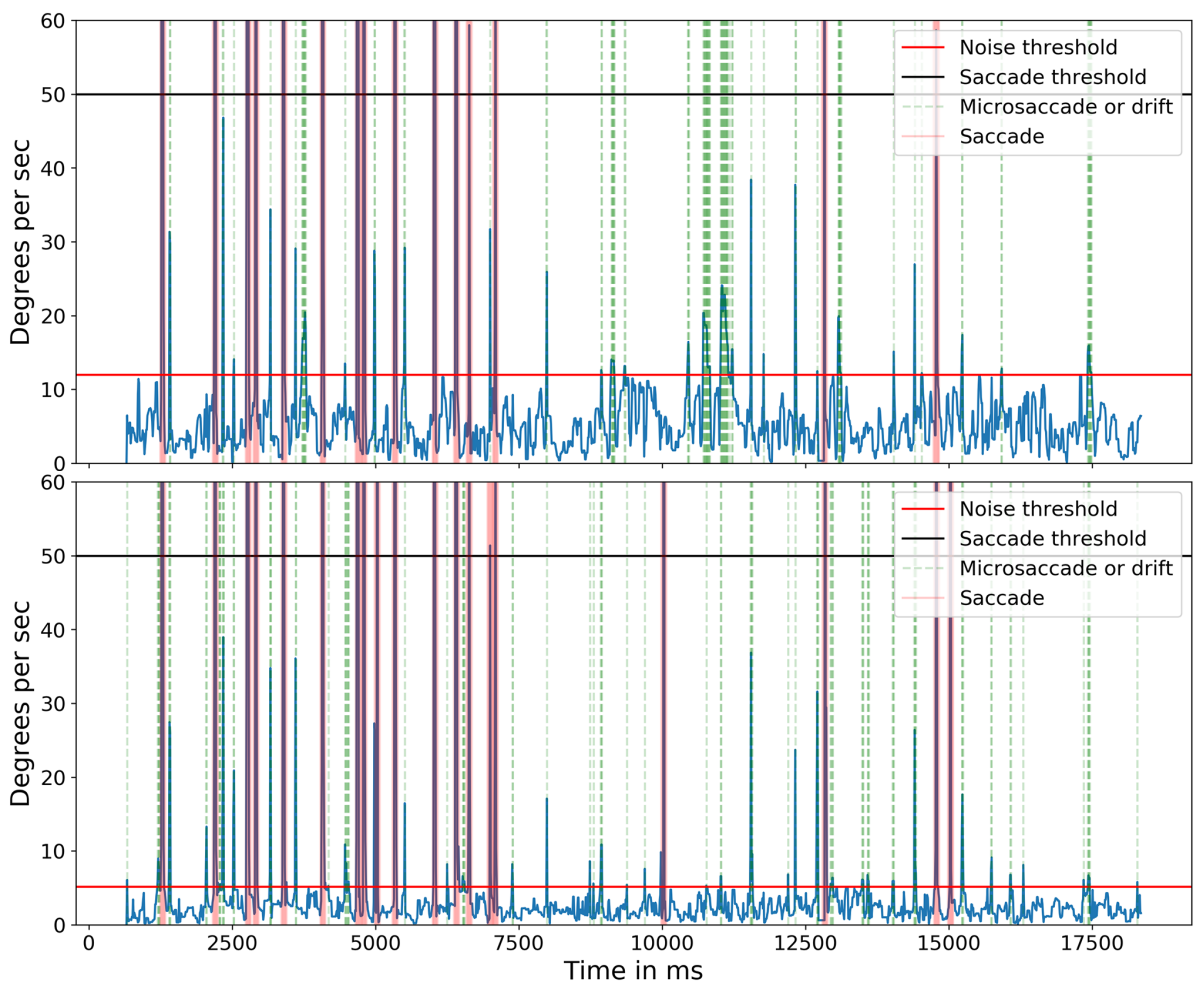
Absolute velocity (deg/sec) of the cyclopean gaze plotted for subject 5 for both without head compensation (top) and after head compensation (bottom). Light green shows the small eye movements detected for their respective threshold parameter.

**Figure 18.**
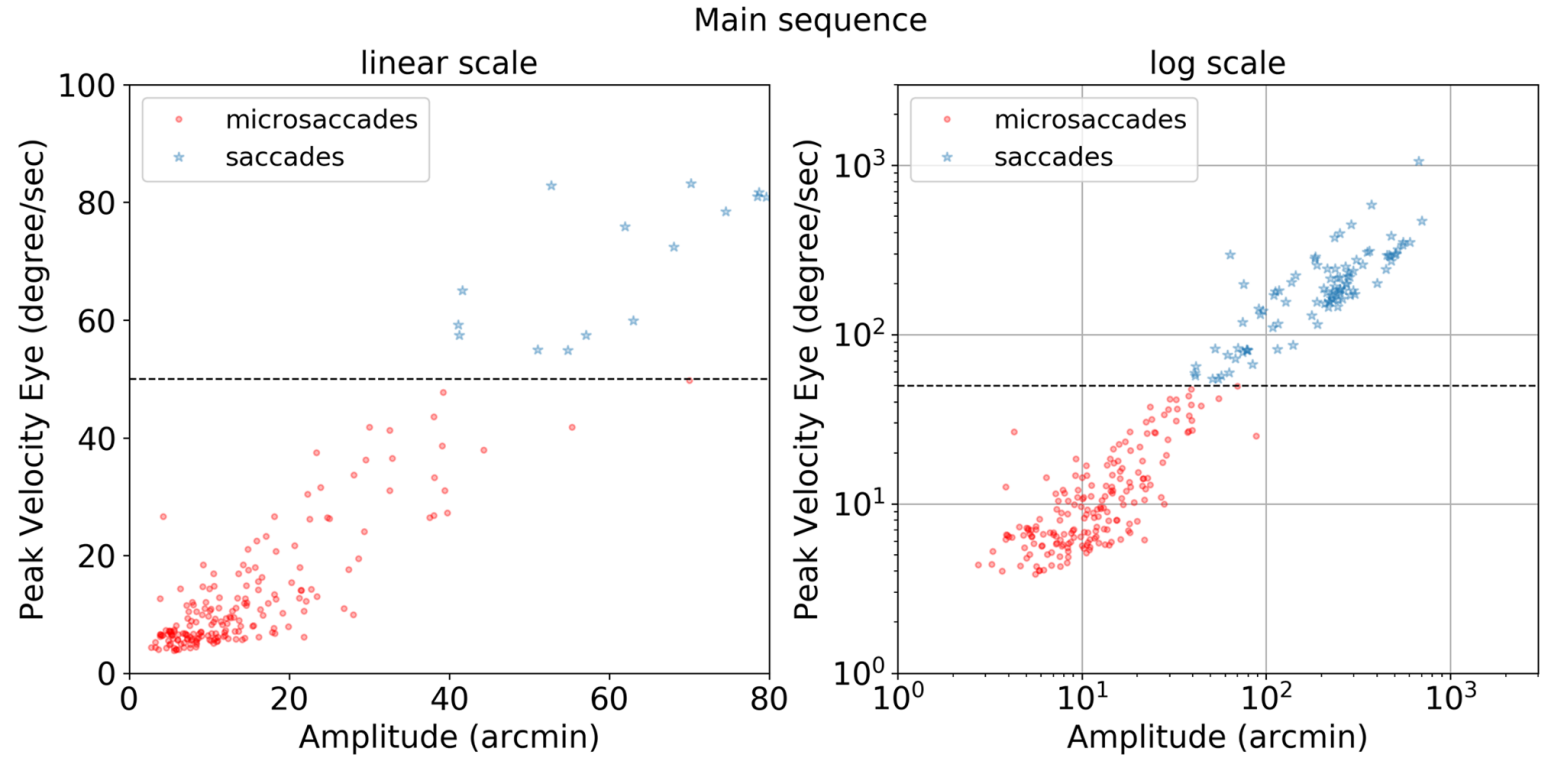
Main sequence plot of peak velocity (degree/sec) vs amplitude (arcmin) in linear and log scales for microsaccades (red circles) and saccades (blue stars). The horizontal dotted line at 50 deg/sec is a fixed threshold used to differentiate saccades and microsaccades.

The importance of compensating for small head movements can be seen in Figure 17. Without compensation (top panel), a threshold of 12.0 deg/sec was necessary to exclude noise. After compensation (bottom panel), a threshold of 5.1 deg/sec allowed microsaccades to be detected more accurately. The one false alarm detected for the compensated data was also detected in the uncompensated data indicating a false alarm resulting from large head motion. Without compensating for head motion, a total of nine microsaccades were missed. Figure 18 shows the ‘main sequence’ (peak velocity vs. amplitude) for microsaccades and saccades detected by our method. Both saccades and microsaccades fall on the main sequence with a slope of 47 s ^-1^ . Microsaccades are defined in this context as all movements with a peak velocity <50 deg/sec.

### Video Stimuli

**Figure 19.**
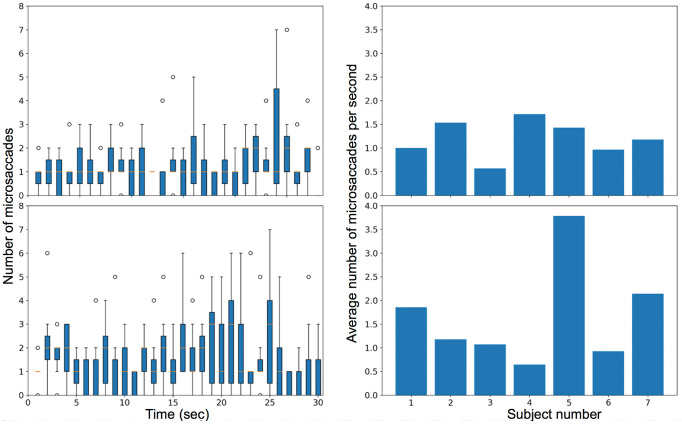
Plot for the rate of microsaccades during trial (left) and the average microsaccades rate for each subject in their overall video (right) when subjects were watching a video of a moving car (top) and watching a video of a shell game (bottom).

In the first video stimulus task, the observers were instructed to fixate on a moving car, leading to smooth pursuit, fixations, saccades, and microsaccades. The box plot with the number of microsaccades detected per second and the variation of the rate of microsaccades among different subjects is plotted for all subjects in Figure 19 (top row). The median number of microsaccades in the video is approximately one per second for most of the trial.

**Figure 20.**
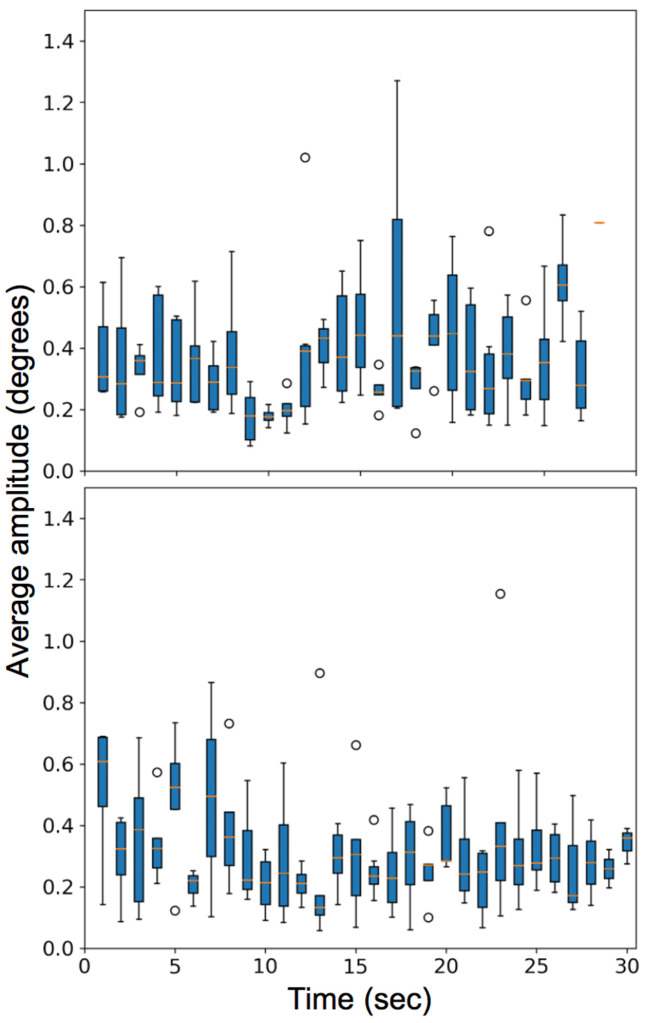
Plot for the average amplitude of microsaccades (degrees) made by various observers for two video stimuli task. (Top) is for watching a video of a moving car and (Bottom) is for watching a shell game.

In the second video task, the observers viewed a ‘shell game’ with three cups and one marble and were instructed to follow the cup that contained the marble. The visual task induced smooth pursuit, saccades, fixations, and microsaccades. The box plot with the number of microsaccades detected per second and the variation of the rate of microsaccades among different subjects is plotted for all subjects in Figure 19 (bottom row). Figure 20 compares the average amplitude of the microsaccades over the entire video for the two video tasks.

## Discussion

Current video-based eye-tracking methods can lead to ambiguities and potential errors when tracking very small eye movements. Our work represents an advance in analyzing microsaccades by tracking iris textures using a high frame rate, high-resolution camera. We segmented the iris from the video frame with a trained CNN and measured the model accuracy using an IoU metric that rewards correct matches and penalizes false matches. The IoU metric was appropriate because we calculated frame-to-frame velocity based on the geometric median of the population of iris feature matches rather than relying on extracting the precise iris boundary. Our CNN model was able to generalize for a varying set of data, though some labeling errors (e.g., eyelids covering the iris) resulted in inconsistencies. The CNN was trained with labels from a single human labeler and might be improved further if labels with multiple-labeler agreement were used for training.

Head motion impacts the number of detectable microsaccades, so we introduced a head compensation model based on simple planar transformations. The model achieved excellent performance with a rigid head model, reducing the overall MSE from 3.59 pixels from the initial reference frame to just 0.42 pixels with standard deviation decreasing to 1/8 ^th^ of the original value.  The model also improved the detection of microsaccades for human subjects as the number of false alarms decreased significantly after compensating for head motion. However, some false alarms remained in cases where a large jitter was observed because of transformation issues (warping). For some subjects, head movements were comparatively large causing more noise in the signal resulting in higher thresholding value obtained from GMMs for microsaccade detection. The current implementation can miss some microsaccades if they occur while updating to a new reference keyframe. When a new reference keyframe was updated there was usually a significant apparent movement, and the motion was misinterpreted as a saccade. Since our major concern lies in detection of microsaccades when a person fixates the instructed targets at a regular interval (approx. 1 sec) and we did not wish to include post-saccadic oscillations or movements compensating for overshoots or undershoots as microsaccade events, we required a minimum period of 52ms (5 frames) between detected velocity peaks. In our tests, one microsaccade was missed during that time interval as the microsaccade timing was aligned to the timestamp of keyframe update.

We have demonstrated the robust detection of microsaccades by tracking the velocity signal generated from iris textures, extracting microsaccades with GMM-based adaptive velocity threshold parameter with a mean of 5.48 deg/sec (σ=1.16). In four of 21 trials in three tasks, the threshold value used was the lower bound of 3.84 deg/sec. Note that for head-fixed data [ 26 ] referred to a threshold of 10 deg/sec as a relatively strict criterion and used the threshold value of 5 deg/sec to validate some monocular events. Our algorithm makes it possible to use this range of parameters (>3.84 deg/sec) consistently without significantly impacting the false alarm rate. 100% of the voluntarily generated small movements over 0.2 degree were detected using our algorithm, and even microsaccades with an amplitude of 0.15 degrees were detected over 73% of the time (41 out of 56 events). Further, microsaccades as small as 0.09 degree were recognized in low-noise backgrounds.

Manually inspecting the video showed that the number of microsaccades was less than the number of instructed targets for the smallest (0.15 degree) microsaccades. It is possible that not all instructed eye movements were actually made; note that an entire grouping of characters fell within the fovea. For three out of seven subjects, all of the 0.15° microsaccades that were identified via visual inspection of the video record were also detected by the algorithm. For one subject, two microsaccades that were not detected by the algorithm were observed to be smaller than the remaining microsaccades for the same observer, perhaps because the preceding microsaccades ‘overshot’ the previous character. It is likely that individual fixations fall on different positions within each character, and some of the characters subtended a horizontal angle of only 0.04 degrees, so it is possible that the individual microsaccades were less than 0.15-degrees if the eye movements were not between character centers. Thus, the actual magnitude of the microsaccades were not exactly the expected value of 0.15, 0.28 and 0.32 degrees calculated from the original target angles. Note too that our algorithm is best suited for event detection rather than providing an exact estimate of position.

Figure 16 (second column) showed one of the sample events where the two eyes moved in opposite directions. This could be the result of true uncorrelated motion (such as vergence movements) or errors in the head compensation model. Vergence was detected by our method especially before and after the blinks as the eyes converged before a blink and diverged after.

The results for our study for the number of microsaccades per second for the two video stimuli tasks are in agreement with Martinez-Conde et al. [ 17 ] and the number and amplitude range are also consistent with the natural scene, picture puzzle and Where's Waldo head-fixed experiments conducted by Otero-Millan et al. [ 24 ] .

Analysis of the video *figure-8* car stimulus suggest that the person is making more microsaccades in the later part of the video. This may be because the car was closer to the camera at that point and the number of catch-up saccades less than 50 deg/sec is expected during smooth pursuit [ 60 ] . We also observe that a high variance of movement is made at the 16th and 24th sec where the car makes a rapid movement and covers approximately 1/6th of the visual field while during the rest of the video the car mostly covers approximately 1/15th of the visual field. During the 13th sec, a long pursuit is expected, and we observed a variation in the number of microsaccades detected since some of the subjects made a large amplitude microsaccade, and few made saccades (>50 deg/sec) in that time interval. In the shell game, it was observed that the number of microsaccades increased from the 11th to the 25th-sec interval even when the glass was at rest perhaps because the person was tracking other objects (hand) in the scene that were still moving.

In the video tasks, the threshold was set for each trial by a GMM. As the smooth pursuit velocity induced by the moving targets was less than the microsaccade velocity threshold, the static GMM was sufficient. In the future, we will implement a continually variable adaptive-threshold in which the GMM will be based on a sliding window so that microsaccades superimposed over higher velocity smooth pursuit can be reliably detected without increasing false alarms during the pursuit.

While the Snellen chart offers a convenient target to induce microsaccades, it presents several challenges. At this scale, several targets fall well within the fovea, so an observer doesn’t need to make saccades to read each character. In addition, significant undershoots and/or overshoots could occur that would make it difficult to identify individual eye movements, without affecting an observer’s ability to read each character. We used the finite interval in an attempt to reduce errors for localizing individual eye movements, but it is still possible that we were not able to accurately parse each microsaccade perfectly.

This experiment could be improved by using a high-resolution digital display in place of the printed Snellen target, and display only one character at a time. This would simplify the identification of each microsaccade by synchronizing presentation and detection times. A higher frame-rate camera could also improve performance. The temporal resolution of the camera impacts the estimate of the amplitude, as it is obtained by integration of velocity over time. With more discrete velocity samples during brief microsaccades we can estimate the amplitude of microsaccades more accurately. Additionally, the signal-to-noise ratio both in the head compensation model and the iris feature tracking method can be improved by using a higher frame rate camera with all-intraframe (ALL-I) encoding. We are also exploring hybrid algorithms that merge velocity and position signals to gain the precision benefit of the iris motion tracking while maintaining the traditional positional information.

In summary, the main contributions of this paper are the use of trained CNNs to provide a more robust solution for iris segmentation across observers with different iris and skin pigmentation; an image-based model for head motion compensation using planar transformations which can be applied in various applications like video stabilization; extracting high quality iris images rich in textures; and an algorithm to reliably detect small eye movements over 0.2 degrees with very high confidence by extracting motion signals with a high signal-to-noise ratio by computing motion distributions rather than relying on precise pupil boundary localization as in pupil and Pupil-CR systems. This method can identify even smaller movements if the velocity of the movement is higher than the threshold parameter (>3.84 deg/sec).

### Ethics and Conflict of Interest

The author(s) declare(s) that the contents of the article are in agreement with the ethics described in http://biblio.unibe.ch/portale/elibrary/BOP/jemr/ethics.html.

JBP is co-inventor of the base method used to extract velocity signals from iris texture features [ 33 ] .

### Acknowledgements

The authors wish to acknowledge the helpful discussions with Michele Rucci and Martina Poletta, who proposed the use of the Snellen target to initiate microsaccades; Emmett Ientilucci who offered valuable guidance and assistance in radiometric measurements; Christye Sisson who shared her expertise on extracting textured iris images; and Anjali Jogeshwar who provided enthusiastic support throughout.
